# Cocaine: A Provoking Risk Factor in Venous Thromboembolism

**DOI:** 10.7759/cureus.6520

**Published:** 2019-12-31

**Authors:** Daniel Griffin, Suji Cha

**Affiliations:** 1 Pulmonology and Critical Care, University of Missouri, Kansas City, USA; 2 Medicine, Kansas City University of Medicine and Biosciences, Kansas City, USA

**Keywords:** pulmonary embolism, cocaine, venous thromboembolism, provoked

## Abstract

Cocaine is a highly addictive recreational drug that is a well-known cause of a variety of disease processes such as stroke, myocardial infarction, and even sudden cardiac death. In current literature, venous thrombosis secondary to cocaine abuse remains under-examined, while the harmful effects of the drug within the arterial vasculature are well-studied and understood. Our case presents a patient who was found to have a large pulmonary embolism and pulmonary infarction after several days of cocaine abuse. This report serves to raise awareness of a potentially life-threatening effect of this drug and to encourage prompt diagnosis and treatment of cocaine-induced pulmonary embolism.

## Introduction

Cocaine is a widely used recreational substance that is responsible for a large number of drug-related adverse events, most commonly within the cardiovascular system. These are not limited to myocardial infarction, aortic dissection, heart failure and cardiomyopathies, stroke, hypertension, arrhythmias, and sudden death [1]. Therefore, it is not surprising that among emergency room visits that involve illicit drugs, cocaine was found to have one of the highest rates of involvement [2].

At this time, the pathophysiology behind cocaine-induced prothrombotic states has yet to be completely elucidated. A commonly proposed mechanism is that cocaine use causes a catecholamine surge, which leads to endothelial damage and downstream prothrombotic effects, including increased levels of fibrinogen and von Willebrand factor in the circulation [3-4]. Without compensatory increased levels of fibrinolysis, these events may promote platelet activity and aggregation and the subsequent formation of blood clots, which can present itself in the form of a deep venous thrombosis or even a fatal pulmonary embolism [5-6]. This report serves to raise awareness of a potentially life-threatening effect of cocaine and to encourage prompt diagnosis and treatment of cocaine-induced pulmonary embolism.

## Case presentation

A 54-year-old male presented to the emergency department with an acute onset of chest pain and shortness of breath. He stated his symptoms started the evening prior and were most notable to his right side. The pain was worse with inspiration and radiated to his right shoulder. He denied any recent illnesses, cough, wheezing, fever, chills, trauma, or difficulty breathing prior to the previous evening.

**Figure 1:**
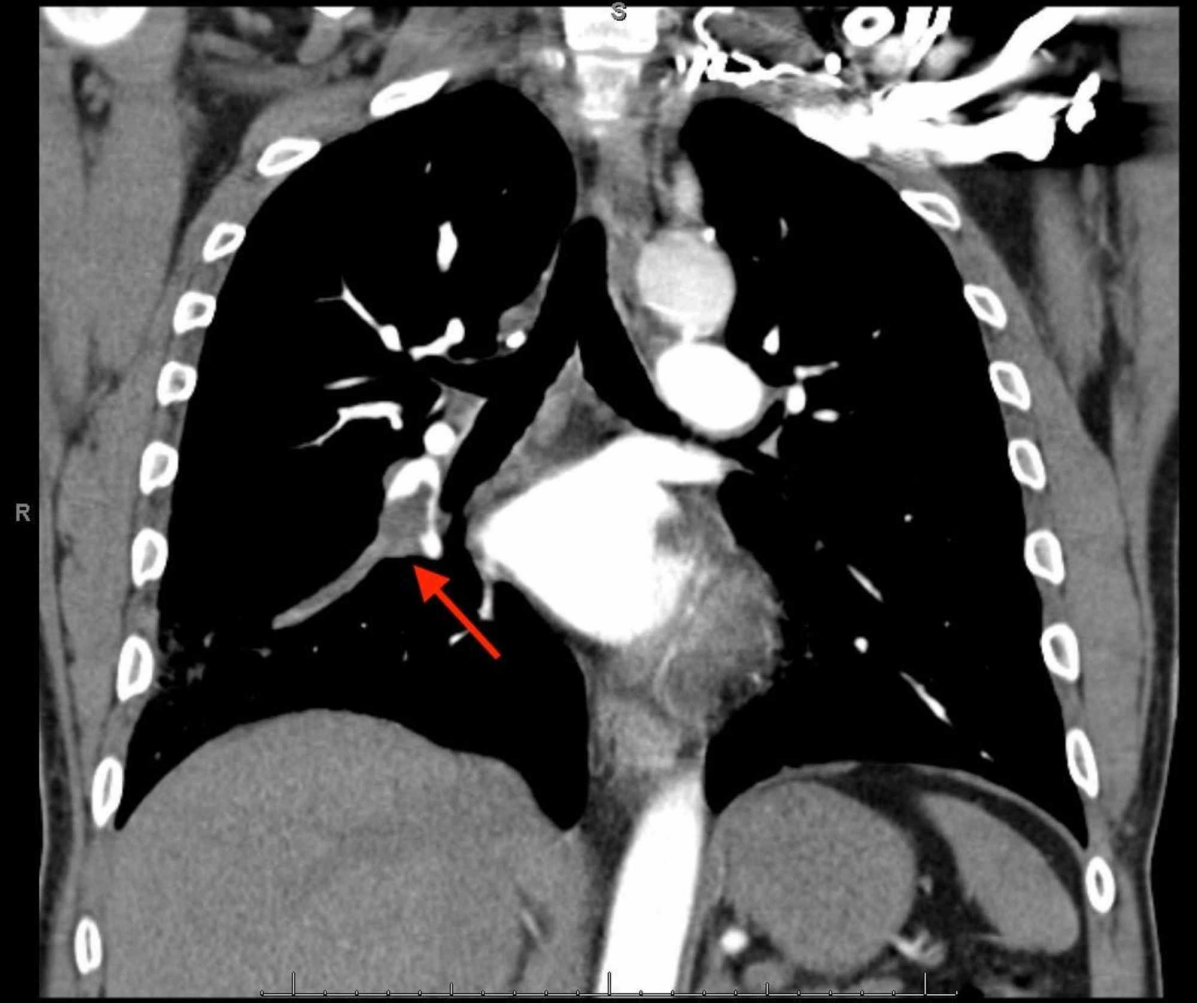
Right-sided Pulmonary Embolism

**Figure 2:**
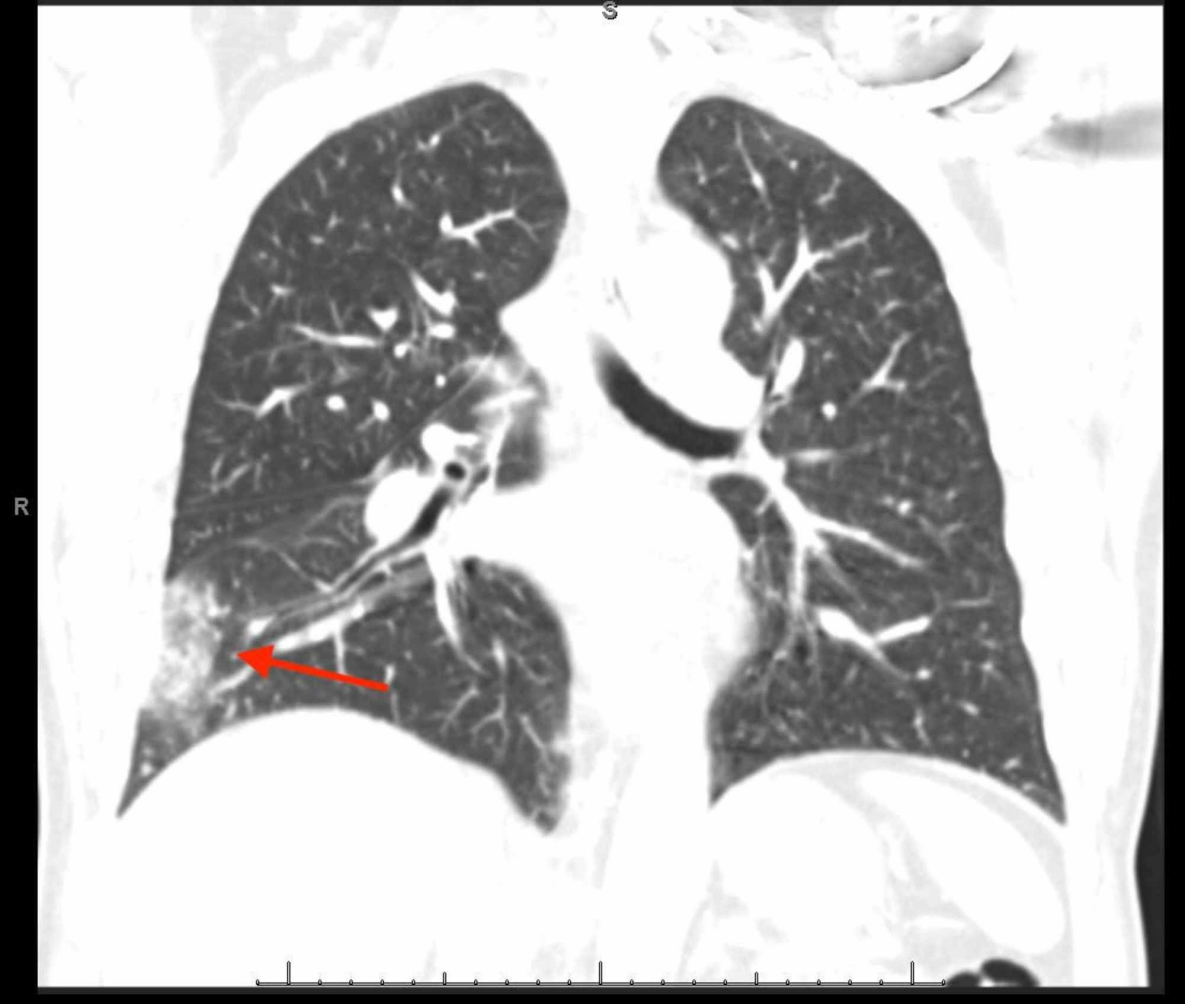
Right-sided Pulmonary Infarct

While in the emergency department, he appeared to be in pain. Vital signs showed a temperature of 98.4 degrees Fahrenheit, heart rate of 77 beats per minute, respiratory rate of 16 breaths per minute, blood pressure of 161/83 mmHg, oxygen saturation of 96% on room air. The physical exam noted rhonchi to the right base but was otherwise unremarkable. Laboratory data noted a normal white blood cell (WBC) count at 8.60 103/cmm (reference range: 4.3 - 10.8103/cmm), troponin <0.01 ng/mL (reference range: 0 - 0.03 ng/mL) and an elevated d-dimer at 1390 ng/mL (reference range: 0 - 500 ng/mL). A urine drug screen was positive for cocaine. Electrocardiogram noted normal sinus rhythm with no acute ST-T wave changes. The patient subsequently underwent a chest X-ray, which noted a right heterogeneous opacity. Gallbladder ultrasound noted no cholelithiasis - computed tomography with angiography, which noted an acute right-sided pulmonary embolism with associated right-sided pulmonary infarction (Figures 1-2). Bilateral lower extremity venous Doppler was negative for deep vein thrombosis.

On further discussion with the patient, he denied any recent trauma or surgeries. He denied any family history of venous thromboembolism. He had not had any change in his activity level as of recent and denied any prolonged car rides or recent air travel. He did admit to using cocaine for the last several days before his admission. Past medical history was notable for coronary artery disease status post bypass grafting approximately one year prior, type 2 diabetes mellitus, hypertension, and hyperlipidemia. Past surgical history was remarkable for coronary artery bypass grafting, which was greater than one year prior, as well as previous back surgery, which was in the distant past. Social history was notable for tobacco use as well as cocaine use, as mentioned above. Family history was significant for coronary artery disease and diabetes. 

During the patient's hospital stay, an echocardiogram was performed and noted a depressed left ventricular ejection fraction of 40% with global hypokinesis. The right ventricle was of normal size and systolic function. There was mild tricuspid regurgitation with an estimated pulmonary artery pressure of 34 mmHg. All findings were similar to the patients' previous echocardiogram findings. He was placed on intravenous heparin and transitioned to rivaroxaban before discharge. On outpatient follow up, he continued to take his rivaroxaban as prescribed and remained free of all cocaine use. Rivaroxaban was continued for a total of three months for a provoked pulmonary embolism secondary to cocaine use.

## Discussion

This case presented a 54-year-old male who developed an acute pulmonary embolism. Other than underlying chronic diseases, he had no predisposing risk factors. He denied a personal or family history of venous thromboembolism; he denied any recent trauma or prolonged travel, and he had been in his usual state of health over the last several months. He did admit to a recent history of significant cocaine use after a prolonged period of abstinence. 

The vascular effects of cocaine have been well documented, but most of these effects seem to center around the arterial complications of cocaine use [1]. We also know that cocaine use results in a more rapid clot formation, increased platelet activation as well as increased activity of plasma plasminogen activator inhibitor (PAI-1), which is associated with increased thrombogenesis [4,7-8]. This data, along with growing case report data of patients experiencing venous thromboembolism in the setting of cocaine use, suggest a possible association [9]. More data will need to be collected to see if the association between cocaine and venous thromboembolism is genuinely causal.

## Conclusions

Cocaine use has long been known to lead to significant arterial thrombi. Currently, there is growing data as well as laboratory data that suggest cocaine leads to both arterial and venous thrombosis. With this growing evidence, the use of cocaine should be considered a provoking risk factor for the development of venous thromboembolism.
